# Effect of an institutional development plan for user participation on professionals' knowledge, practice, and attitudes. A controlled study

**DOI:** 10.1186/1472-6963-11-296

**Published:** 2011-11-02

**Authors:** Marit By Rise, Hilde Grimstad, Marit Solbjør, Aslak Steinsbekk

**Affiliations:** 1 Department of Public Health and General Practice, Norwegian University of Science and Technology, Trondheim, Norway

## Abstract

**Background:**

Governments in several countries attempt to strengthen user participation through instructing health care organisations to plan and implement activities such as user representation in administrational boards, improved information to users, and more individual user participation in clinical work. The professionals are central in implementing initiatives to enhance user participation in organisations, but no controlled studies have been conducted on the effect on professionals from implementing institutional development plans. The objective was to investigate whether implementing a development plan intending to enhance user participation in a mental health hospital had any effect on the professionals' knowledge, practice, or attitudes towards user participation.

**Methods:**

This was a non-randomized controlled study including professionals from three mental health hospitals in Central Norway. A development plan intended to enhance user participation was implemented in one of the hospitals as a part of a larger re-organizational process. The plan included i.e. establishing a patient education centre and a user office, purchasing of user expertise, appointing contact professionals for next of kin, and improving of the centre's information and the professional culture. The professionals at the intervention hospital thus constituted the intervention group, while the professionals at two other hospitals participated as control group. All professionals were invited to answer the Consumer Participation Questionnaire (CPQ) and additional questions, focusing on knowledge, practice, and attitudes towards user participation, two times with a 16 months interval.

**Results:**

A total of 438 professionals participated (55% response rate). Comparing the changes in the intervention group with the changes in the control group revealed no statistically significant differences at a 0.05 level. The implementation of the development plan thus had no measurable effect on the professionals' knowledge, practice, or attitudes at the intervention hospital, compared to the control hospitals.

**Conclusion:**

This is the first controlled study on the effect on professionals from implementing a development plan to enhance user participation in a mental health hospital. The plan had no effect on professionals' knowledge, practice, or attitudes. This can be due to the quality of the development plan, the implementation process, and/or the suitability of the outcome measures.

## Background

User participation in health care is highly emphasized in the western world [1-3]. In several countries, e.g. in Scandinavia [4] and UK [5], governments attempt to strengthen participation through guidelines and law regulations, making health care organisations responsible for enhancing user participation in their service. Planning and implementing activities to meet these requirements, such as having user representation in administrational boards, improving information to users, and enhancing user participation in clinical work thus become a part of the responsibilities and daily tasks in health organisations. The health care organisations' goals will subsequently be put into practice by those who are providing the service; the professionals. Studies have shown that collaboration and partnership between service users and providers are essential in the process of participation [6], that such collaboration can be challenging [7-9], that participation is more common at the individual level than the organisational level [10], and that exploring professionals' attitudes towards user participation is important [8]. The professionals are thus vital when enhancing user participation in health care organisations [6,11].

There exists many well-funded definitions of user participation [5,12-18]. The concept has been described as dialogue [12,15], as a change of the patient role from passive to active [13], as ways the public can influence the policy and health services [13,14], as users making use of their experience [14,19], and as a right for patients to be involved in decision making [16,17]. There are also several proposed benefits from user participation in health care; extending services and making them more accessible, improving information [1], reducing medical errors, improving patient safety [2], improving the coordination of care in chronic diseases [20], improving clinical decision making [21], and improving alignment of patients' and physicians' goals and agendas [22]. Several studies have discussed potential challenges and hindrances towards user participation in health care. Some have found differences between professionals and patients regarding experiences from participation [23,24], the understanding and commitment to participation [6,12,25,26], and the extent of user participation [26]. Other barriers described are restraints of time and resources [26]. Although collaboration between users and professionals are important, some studies have shown that professionals in health services can hold negative attitudes towards user participation [27-29].

Several studies have investigated how and to what extent user participation is conducted in health services, including professionals' knowledge and attitudes [10,30-33]. Research reviews show, however, that there is a limited number of controlled (randomized and non-randomized) studies investigating effects [34]. One study, investigating an intervention program to enhance user participation among professionals in an inpatient setting, showed impact on organisational user participation patient collaboration, and carer involvement [35]. Besides this, there exists a lack of controlled studies on the effect on professionals from implementing an extensive and comprehensive development plan for enhancing user participation in health care organisations.

The implementation of the development plan was intended to enhance user participation in the hospital. Since professionals are vital for carrying out user participation in collaboration with users, it would be reasonable to anticipate that the organisational changes should have a positive impact on the professionals' knowledge, practice, and attitudes towards user participation. In addition, the comparison of any change with a control group would ensure that any impact was due to the development plan.

The aim of this study, therefore, was to study whether implementing a development plan intending to enhance user participation in a mental health hospital had any effect on the hospital professionals' knowledge, practice, or attitudes towards user participation.

## Methods

### Study design and ethics

This was a non-randomized controlled study including professionals from three mental health hospitals in Central Norway. One of the hospitals (intervention hospital) implemented a development plan to enhance user participation, and two hospitals participated as control group. A non-matched sample of professionals from the three hospitals filled out the same questionnaire two times, 16 months apart. The aim was to compare the change in the intervention hospital with the control hospitals. The study took place from November 2008 to December 2010, and was approved by the regional committee for medical and health research ethics in Central Norway, the Norwegian Data Inspectorate, and the hospitals' management.

### Setting

The three hospitals are part of the same hospital trust, and include all district psychiatric centres in one of the trust's main hospitals. The intervention hospital covers a catchment area of 96.000 persons, with urban and semi rural areas including parts of a large Norwegian city. The two control hospitals cover catchment areas of 74.000 and 47.000 persons respectively, with urban, semi rural, and rural areas including parts of the above-mentioned city. The three hospitals provide the same types of service; in-patient treatment (5.4 beds per 10.000 inhabitants), out-patient treatment, and ambulatory services. The intervention hospital was relocated and reorganised in January 2009. Several units were merged and co-localised, and an ambulatory acute treatment team was established. The reorganisation was based on an over-arching plan focusing on professional development and improvement of collaboration and patient flow. The reorganizational plan also included the intervention in this study; a development plan for user participation.

### Intervention

As part of the structural reorganization and relocation, the intervention hospital formulated and approved a development plan for user participation. The development plan was formulated by a project group, constituted by administrators, health professionals, and user representatives (recruited from mental health user organisations), working from fall 2007 to June 2008. The plan was thus based on clinical experience and knowledge, and the hospital administration's aim for the health services they provide. The final plan was approved in the health care trust in June 2008.

The development plan included several initiatives to enhance user participation both on a system and an individual level (table 1). The initiatives in the plan were chosen by the project group based on their knowledge about user participation, and the services they wanted to provide after the reorganisation. In April 2010, fifteen months after the implementation started, most of the initiatives were completed, while others were not. Information on the implementation status at this time was collected through interviews with key professionals and documents produced by the hospital. The implementation status in April 2010 described in this article was confirmed by the hospital's management.

**Table 1:** Initiatives in the development plan

Planned initiatives in development plan sanctioned in June 2008 and planned implemented from January 2009	Status for implementation in April 2010
Establishing a patient education centre	A patient education centre was established in November 2009, and employed two persons. A user representative participated in the planning and starting of the centre, and representatives partake in the daily work.
Establishing an office run by users where various user representatives shall be available to the users of the centre	An office and information centre for users was established in January 2010. The office provides information material, telephone and Internet for patients and next of kin. Two user organisations and representatives from the regional labour and welfare administration use the office weekly.
Purchasing user expertise up to 17.5 hours per week	The centre's budget allows for buying up to 17.5 hours of user expertise per week, but normally buys 10-12 hours per month. A user representative is employed 20% for the research project on self administered places/beds.
Establishing a strategy for education of user representatives	Not implemented. Education of user representatives has been assigned to the user organisations.
Appointing contact personnel for next of kin in each section	In March 2009 one personnel from each unit has been appointed contact person for next of kin.
Allowing money in the budget for patient education	Money for patient education have since January 2009 been a part of the patient education centre's working budget.
Tentative proceedings with places/beds administered by the patients themselves	A randomized controlled trial on places/beds administered by patients was started in May 2010. One user representative is participating in the steering committee, and two in the research group. User expertise equivalent to 20% employment is bought during this study.
Improving the centre's communication and information materials	A group was established before relocation to evaluate and suggest measures to improve the centre's communication and information materials. The work in this group stopped after a few meetings. Outwards communication has been discussed at several staff meetings during 2009 and 2010.
Formulate and implement a strategy for quality assurance of attitudes and culture among the personnel	Tentative plans were discussed with user representatives in spring 2009. A philosopher was temporarily employed during the fall 2009. He conducted group sessions with health personnel to discuss attitudes towards user participation. The work stopped in 2009. The implementation group (administrators, health personnel and user representatives) discussed attitudes and culture at 6-8 meetings during the implementation process.
Implementing a web based system (Sampro) for collaborating and coordinating individual plans and individual education plans for patients.	An educational course led by an external course supervisor was held for 4 patients and their therapists in April 2010. In one of the in-patient units therapist have received training in using the system, and patients are continuously offered to use this system.
Informing patients; in general about the centre, about their right to change therapist, and about setting treatment goals	Information has been discussed at several meetings in the executive group, but no concrete initiatives have been planned or implemented.
Tentative proceedings with using Client Directed Outcome Informed therapy in out-patient sessions.	A research trial on Client Directed Outcome Informed therapy in out-patient sessions started in February 2010, and is currently running.
(Not in development plan)	The patient education centre reviewed each unit's work with patient education from January 2010, and decided to appoint one contact person for patient education per unit. Per April 2010 6 out of 8 units had contact persons.
(Not in development plan)	To ensure identification of and care for in-patients' children a group in charge was appointed in January 2010.
(Not in development plan)	All in-patient units conduct regular "house meetings" where patients are encouraged to raise issues which are subsequently discussed in management meetings.
(Not in development plan)	Patients and users are represented in the panel overseeing the quality of the services, and are participating in the processes of introducing new service initiatives.

### Implementation

The intervention was implemented from January 2009. The hospital manager was in charge of the implementation process, and the everyday executive responsibility was ensured by one of three unit managers at the hospital. An implementation group consisting of the unit manager, several administrators, health professionals, and user representatives (recruited from mental health user organisations) was established in August 2009 to supervise and follow up the implementation. The group had six meetings until January 2010 when the group was dissolved.

### Participants

All employees including psychiatrists, nurses, psychologists, other health and social workers, and administrative professionals in the three mental health hospitals were invited to participate in this study. They were identified from updated lists including all paid employees registered the current month. Lists of employees were obtained from the hospitals' administrative offices. To maintain anonymity and to invite all employees at both measurement points, the employees participating at the different time points were not matched. The group of employees answering the questionnaire was thus not identical in the first and the second time of measurement. The participants concordantly constituted four independent samples; sample 1 and 2 constituted the intervention group, and sample 3 and 4 the control group. The difference between results in sample 1 and sample 2 was compared with the difference between results in sample 3 and 4.

### Data collection

All employees at the intervention hospital were invited to fill out a questionnaire in December 2008 (before the implementation of the development plan started) and in April 2010 (16 months later). During the same period, all employees at the two control hospitals were invited to fill out a questionnaire two times with a 16 months interval. All rounds of invitations were mailed to all employees. The questionnaires were sent by post to the employees' private address with pre-paid return envelopes that were returned directly to the researchers. One reminder was sent.

### Outcome measures

To measure knowledge, practice, and attitudes towards user participation among the professionals the Consumer Participation Questionnaire (CPQ) was used [30]. The questionnaire was translated to Norwegian for this study. The translation was carried out through a process where two persons independently translated the questions from English to Norwegian, and two persons independently translated from Norwegian to English. Lack of accordance in the translations was discussed until consensus was reached. Effort was made to keep the translated version as similar to the original as possible. The final version was then discussed in a group of researchers, but not piloted or validated further prior to use.

The original CPQ questionnaire includes 20 questions. To elaborate on question no. 8 in the original questionnaire (Does your agency solicit user input for planning of mental health service?), a question no. 8a (Does your agency solicit input from user organisations for planning of mental health service?) was added. Question no. 14 in the original (Should users be involved in the evaluation and diagnosis of their presenting problems?) was split in two questions to ensure interpretable results (no. 14 and 14a).

We also added 8 questions to ensure that most aspects of the professionals' views and practices regarding user participation were included. These questions were formulated, discussed, and refined during meetings in the research group. The questionnaire used in this study thus included a total of 30 questions ranging from no. 1 to 28 (including no. 8a and 8b, 14 and 14a).

The questions were organised into three thematic areas for this study: Twelve of the 30 questions measured professionals' knowledge on user participation, seven measured practice, and 11 measured attitudes. These thematic areas were first identified by the 1^st ^author through looking at each of the questions and grouping them. The grouping was discussed in the research group to ensure face validity. No further validation was conducted.

### Statistical analysis

The results from the two control hospitals were combined. Pearson's chi square tests were used to identify any differences in proportions within the two groups at the two points of measurement. Binary logistic regression was used to analyse changes from baseline to 16 months in the intervention hospital and the control hospitals respectively. Demographic variables with trends for difference (p < 0.1) within each group (intervention group; number of years worked at the unit-control group; category of patients working with, table 2) were added to the time of measurement variable in the regression model. A test of proportion [36] was used to calculate ratio odds ratio (ROR) to compare the effect in the intervention group with the effect in the control group. A significance level of 5% (p < 0.05) was chosen. Analysis was done with SPSS 17.0 for Windows (SPSS Inc., Chicago, IL).

**Table 2:** Demographic variables-comparison of proportions at baseline and follow-up (total N = 438).

Variable	Intervention group			Control group		
	Baseline N = 89*	Follow-up N = 93*	p-value^†^	Baseline N = 133*	Follow-up N = 123*	p-value^†^
*Female*	72.7%	77.2%	0.491	87.0%	85.1%	0.663
*Current position*			0.797			0.223
- Nurse	36.4%	37.8%	(0.845)	28.5%	38.5%	(0.090)
- Medical doctor	4.5%	5.6%	(0.758)	7.7%	8.2%	(0.882)
- Psychologist	21.6%	28.9%	(0.263)	13.8%	15.6%	(0.669)
- Health/welfare worker	25.0%	18.9%	(0.324)	37.7%	28.7%	(0.130)
- Administrative/Other	12.5%	8.9%	(0.435)	12.3%	9.0%	(0.399)
*Leadership responsibility*	12.5%	7.7%	0.285	7.7%	9.0%	0.704
*Category of patients working with*			0.594			0.069
- In-patients	29.5%	26.1%	(0.605)	49.6%	50.4%	(0.900)
- Out-patients	52.3%	50.0%	(0.760)	32.8%	34.1%	(0.823)
- Both	11.4%	18.5%	(0.181)	12.2%	15.4%	(0.455)
- Not working with patients	6.8%	5.4%	(0.699)	5.3%	0%	(0.009)
*No of years worked in this unit. Mean(SD)*	6.4 (6.3)	4.8 (5.5)	0.069^‡^	6.8 (6.2)	7.2 (6.9)	0.631^‡^

Numbers are percentages of total N for each sample unless otherwise stated.

* The N in the four samples varied for each question due to missing answers on the variables (0.8%-3.3%).

^† ^p-value calculated using Pearson's chi square.

^‡ ^p-value calculated using independent samples t-test.

## Results

Eighty-nine of 184 (48%) members of professionals responded at the intervention hospital at baseline (sample 1), and 93 of 179 (52%) responded 16 months later (sample 2). At the control hospitals, 133 of 221 (60%) professionals responded at baseline (sample 3), while 123 of 211 (58%) responded 16 months later (sample 4). The total number of participants was 438, and the total response rate was 55%.

The total sample (N = 438) included 81.5% females, 34.9% nurses, 28.6% health/social workers, 19.1% psychologists, 10.7% administrative/others, and 6.7% medical doctors. 9% of the participants had leadership responsibility. 40.8% worked with in-patients, 40.8% with out-patients, 14.3% worked with both groups, and 4.1% did not work therapeutically with patients. The participants had worked between 0 and 30 years in the unit with a mean of 6.4 years (SD 6.3) and a median of 5.0.

Differences in demographics between baseline and follow-up within each of the two groups are described in table 2. There were no significant differences at a 0.05 level. In the intervention group there was a trend towards professionals at follow-up had worked fewer years at the unit (p = 0.069) than the professionals at baseline. In the control group there was a trend for differences in which category of patients professionals worked with (p = 0.069). These variables were added to the binary logistic regression models for the groups respectively (and were thus controlled for when comparing the changes within and between the groups).

Comparing the distribution in the intervention hospital and the control hospitals at baseline indicated no systematic differences between the groups (proportions at baseline given in table 3 analysis of baseline comparison not shown).

**Table 3:** Professionals' knowledge, practice, and attitudes at baseline and follow-up for intervention and control group.

	Intervention			Control		
Knowledge	Baseline N = 89^1^	Follow-up N = 93^1^	p-value^†^	Baseline N = 133^1^	Follow-up N = 123^1^	p-value^†^
*1. Does your unit have a complaints procedure for users? *(% yes)	47.7%	51.6%	0.601	66.7%	74.0%	0.202
*5. Have you heard or read anything about consumer involvement and participation in the provision of mental health services? *(% yes)	91.0%	91.3%	0.945	88.6%	95.0%	0.065*
*8a. Does your unit solicit input from users for the planning of mental health services? *(% yes)	69.7%	64.5%	0.460	74.2%	71.3%	0.600
*8b. ^# ^Does your unit solicit input from user organisation for the planning of mental health services? *(% yes)	47.2%	52.2%	0.503	36.4%	52.5%	0.010**
*9. Does your unit routinely conduct user satisfaction service on the service it offers? *(% yes)	34.1%	34.4%	0.964	37.9%	44.3%	0.301
*10. Are users involved in the hiring decisions of your unit's staff? *(% yes)	0%	0%	1.00	0%	2.5%	0.070*
*11. Are users invited to participate in staff training meetings at your agency? *(% yes)	3.4%	21.7%	0.000**	6.8%	15.6%	0.026**
*12. Has your unit ever asked users to act as teachers at staff training events? *(% yes)	7.9%	15.1%	0.129	16.8%	26.2%	0.067*
*13. Does your unit sponsor events/forums that educate users about their rights and entitlements? *(% yes)	37.1%	44.1%	0.336	61.1%	59.0%	0.739
*21*. ^# ^*Does the unit have a users' committee? *(% yes)	28.1%	39.1%	0.116	56.5%	54.5%	0.746
*22*. ^# ^*Does the unit have representatives or spokespersons on behalf of the users? *(% yes)	22.5%	31.5%	0.171	51.9%	53.3%	0.827
**Practice**						
*3. Are users told they have a right to see and/or correct their records? *(% yes)	58.4%	71.1%	0.076*	58.3%	62.3%	0.519
*4. Are users informed about the facts about confidentiality and privacy regarding information contained in those records? *(% yes)	79.8%	81.5%	0.766	84.8%	89.4%	0.276
*7. Do you tell users what goals are intended to be accomplished by the treatment? *(% yes)	81.2%	89.8%	0.108	89.1%	95.9%	0.041**
*25*. ^# ^*Do you have enough time to ensure users' participation? *(Yes)	59.3%	60.9%	0.826	67.8%	69.1%	0.827
*26*. ^# ^*In your opinion, are next of kin in general sufficiently involved? *(% yes)	30.1%	28.6%	0.826	25.2%	33.9%	0.137
*27*. ^# ^*How would you describe the collaboration with next of kin in general?*(% very good/quite good)	44.6%	49.5%	0.520	42.3%	46.7%	0.481
*28*. ^# ^*Do you inform users about relevant self-help groups and user organisations? *(% yes)	85.4%	77.9%	0.213	78.4%	82.6%	0.412
**Attitudes**						
*6. In most cases, where does the responsibility for deciding the goals of treatment usually lie?* (% entirely/mostly the user)	6.8%	12.9%	0.172	12.0%	7.3%	0.204
*14. Should users be involved in the evaluation of their presenting problems? *(% always/usually)	87.5%	95.6%	0.050**	90.0%	94.3%	0.211
*14a. Should users be involved in the diagnosis of their presenting problems? *(% always/usually)	80.9%	84.8%	0.488	66.4%	72.4%	0.305
*15. In your opinion, should users contribute to the writing of their notes and records? *(% yes)	43.7%	41.6%	0.778	42.5%	38.0%	0.470
*16. In your opinion, should users be involved in the planning of their own treatment? *(% yes)	97.7%	98.9%	0.528	97.0%	100%	0.052*
*17. How would mental health service change if users were employed by that service? *(% improve)	71.6%	65.5%	0.396	56.2%	63.7%	0.241
*19. How would mental health service change if users were involved in the planning and/or delivery of those services? *(% improve)	90.7%	84.6%	0.220	81.7%	83.9%	0.656
*23*. ^# ^*How would you describe the unit's general attitude towards user participation? *(% quite good/very good)	32.2%	42.4%	0.158	61.8%	64.5%	0.665
*24*. ^# ^*In your opinion, do users understand the information you give about their illnesses and treatment opportunities? *(% yes)	98.8%	95.1%	0.182	95.9%	96.6%	0.783
*18. In your opinion, what are the most important reasons when users of mental health care don't want to be involved?*						
- Too vulnerable (% yes)	36.0%	37.6%	0.814	42.9%	39.8%	0.624
- Lacking in self-confidence (% yes)	59.6%	39.8%	0.008**	60.2%	52.0%	0.191
- Lacking in ability or knowledge (% yes)	12.4%	9.7%	0.563	14.3%	17.9%	0.433
- Lacking in motivation (% yes)	51.7%	38.7%	0.079*	42.1%	43.9%	0.772
- Lack of trust in the ability of the services to provide help (% yes)	38.2%	28.0%	0.142	24.8%	25.2%	0.942
- Not wanting to have any further contact after getting better (% yes)	44.9%	40.9%	0.578	37.6%	39.0%	0.814
- Other reasons (% yes)	16.9%	7.5%	0.054*	18.0%	17.1%	0.838
*20. In your opinion, if users were involved in planning and/or carrying out the mental health service, how would the service develop?*						
- Upgrading of services and delivery (% yes)	73.0%	65.6%	0.277	56.4%	59.3%	0.632
- Less burnout and stress for providers of those services (% yes)	11.2%	12.9%	0.730	9.0%	10.6%	0.677
- More chance that users would benefit from those services the first time round (% yes)	86.5%	75.3%	0.054*	71.4%	71.5%	0.984
- Less chance of the "revolving door" syndrome, where users keep returning with the hope of finding help (% yes)	27.0%	30.1%	0.639	27.1%	22.0%	0.342
- Downgrading of services and delivery (% yes)	1.1%	4.3%	0.190	5.3%	4.9%	0.888
- More burnout and stress for the providers of those services (% yes)	3.4%	7.5%	0.219	6.8%	6.5%	0.933
- That users would only be regarded as tokens by the professionals (% yes)	9.0%	10.8%	0.690	7.5%	6.5%	0.751
- That users would not understand the language used, and therefore find it difficult to give any input (% yes)	3.4%	4.3%	0.744	8.3%	9.8%	0.678
- Other developments (% yes)	7.9%	7.5%	0.932	9.0%	5.7%	0.310

Proportions and test of difference within each group. Numbers are percentages of total N for each sample.

^1 ^N is the no of participants who returned completed questionnaires. The N in the four samples varied for each question due to missing answers on the variables (0%-11%).

*^# ^*Questions marked ^# ^were added to the Consumer Participation Questionnaire (CPQ) in this study.

* p < 0.1, ** p < 0.05,

^† ^p-value calculated using Pearson's chi square test.

### Changes within the groups

The distribution of answers and the changes in proportions from baseline to follow-up for both the intervention group and control group are presented in table 3.

#### Knowledge about user participation

At the intervention hospital there was significant change at a 0.05 level in two out of eleven questions; "Users invited to participate in professionals training meetings" (no. 11, AdjOR = 11.6, p = 0.001) and "Users asked to act as teachers at professionals training events" (no. 12, AdjOR = 3.0, p = 0.046). At the control hospitals there was significant change at a 0.05 level in two out of eleven questions; "Input solicited from user organisation for the planning of services" (no. 8b, AdjOR = 1.9, p = 0.014) and "Users invited to participate in professionals training meetings" (no. 11, AdjOR = 2.7, p = 0.030). There were no trends for change (p < 0.1) in either of the groups. There were thus only small changes from baseline to follow-up in the professionals' knowledge about user participation within either of the groups.

Looking for any direction of change from baseline to follow-up within the intervention hospital it was found an AdjOR above 1.0 (increase in knowledge) in 9 out of 10 questions (p = 0.001-0.667). Within the control hospitals we found an AdjOR above 1.0 (increase in knowledge) in seven out of 10 questions (p = 0.014-0.949).

#### Practice of user participation

There were no significant changes from baseline to follow-up in the professionals' practice of user participation within either of the groups. There were trends for change (p < 0.1) in two questions in the intervention group; "Users are told they have a right to see and/or correct their records" (no. 3, AdjOR = 1.8, p = 0.072) and "Users are told what goals are intended to be accomplished by the treatment" (no. 7, AdjOR = 2.2, p = 0.094). There were no trends for change from baseline to follow-up in the control group. Thus, there was no significant change from baseline to follow-up in the professionals' practice of user participation within either of the groups.

Looking for any direction of change from baseline to follow-up within the intervention hospital we found an AdjOR above 1.0 (more participation) in four out of seven questions (p = 0.072-0.660). In the control hospitals we found an AdjOR above 1.0 (more participation) in six out of seven questions (p = 0.172-0.890).

#### Attitudes towards user participation

There were no significant changes from baseline to follow-up regarding the professionals' attitudes towards user participation within either of the groups. There was a trend for change within the intervention group in one question; "Users should be involved in the evaluation of their presenting problems" (no. 14, AdjOR = 3.0, p = 0.073). There were no trends for change in the control group.

Looking for any direction of change from baseline to follow-up within the intervention hospital we found an AdjOR above 1.0 (improved attitudes towards user participation) in six out of 9 questions (p = 0.072-0.660). At the control hospitals we found an AdjOR above 1.0 (improved attitudes) in six out of 9 questions (p = 0.172-0.627).

The changes from baseline to follow-up in the intervention hospital and the control hospitals respectively are presented in table 4 (the first two columns).

**Table 4:** Comparison of changes within and between the groups.

	WITHIN				BETWEEN	
	Intervention N = 182^1^		Control N = 256^1^		Intervention vs. Control	
Variable	AdjOR (95% CI)	p-value^†^	AdjOR (95% CI)	p-value^†^	Ratio OR (95% CI)	p-value^†^
**Knowledge**						
*8b. ^# ^Does your unit solicit input from user organisation for the planning of mental health services? *(% yes)	1.3(0.7-2.4)	0.390	1.9(1.1-3.2)**	0.014	0.7(0.3-1.5)	0.334
*11. Are users invited to participate in staff training meetings at your unit? *(% yes)	11.6 (2.6-52.3)**	0.001	2.7(1.1-6.4)**	0.030	4.4(0.8-25.0)*	0.098
*12. Has your unit ever asked users to act as teachers at staff training events? *(% yes)	3.0(1.0-8.8)**	0.046	1.6(0.9-3.0)	0.116	1.8(0.5-6.3)	0.340
*21. ^# ^Does the unit have a users' committee? *(% yes)	1.6(0.9-3.1)	0.130	1.0(0.6-1.6)	0.949	1.7(0.7-3.8)	0.220
*22. ^# ^Does the unit have representatives or spokespersons on behalf of the users? *(% yes)	1.7(0.8-3.3)	0.142	1.2(0.7-2.0)	0.531	1.4(0.6-3.4)	0.418
**Practice**						
*3. Are users told they have a right to see and/or correct their records? *(% yes)	1.8(0.9-3.4)*	0.072	1.1(0.7-1.9)	0.627	1.6(0.7-3.6)	0.270
*7. Do you tell users what goals are intended to be accomplished by the treatment? *(% yes)	2.2(0.9-5.4)*	0.094	2.2(0.7-6.6)	0.176	1.0(0.2-4.2)	0.995
*26. ^# ^In your opinion, are next of kin in general sufficiently involved? *(% yes)	0.8(0.4-1.7)	0.640	1.5(0.8-2.6)	0.172	0.6(0.2-1.4)	0.221
**Attitudes**						
*6. In most cases, where does the responsibility for deciding the goals of treatment usually lie? *(% entirely/mostly the user)	1.8(0.6-5.1)	0.281	0.5(0.2-1.3)	0.152	3.3(0.9-13.0)*	0.082
*14. Should users be involved in the evaluation of their presenting problems? *(% always/usually)	3.0(0.9-10.2)*	0.073	1.8(0.7-4.8)	0.233	1.7(0.4-7.9)	0.514
*23. ^# ^How would you describe the unit's general attitude towards user participation? *(% very/quite good)	1.6(0.9-3.1)	0.121	1.2(0.7-2.0)	0.605	1.4(0.6-3.3)	0.393
*18. In your opinion, what are the most important reasons when users of mental health care don't want to be involved?*						
- Lacking in self-confidence (% yes)	0.5 (0.3-0.9)**	0.026	0.6 (0.4-1.1)*	0.085	0.8 (0.4-1.7)	0.550
- Lacking in motivation (% yes)	0.7 (0.3-1.1)	0.123	1.0 (0.6-1.7)	0.977	0.7 (0.3-1.5)	0.315
- Lack of trust in the ability of the services to provide help (% yes)	0.6 (0.3-1.2)	0.169	0.9 (0.5-1.6)	0.761	0.7 (0.3-1.7)	0.416
- Other reasons (% yes)	0.4 (0.2-1.1)	0.076	0.9 (0.5-1.8)	0.793	0.5 (0.1-1.5)	0.188
*20. In your opinion, how would the service develop if users were involved in planning and/or carrying out the mental health service?:*						
- More chance that users would benefit from those services the first time round (% yes)	0.6 (0.3-1.3)	0.178	0.8 (0.4-1.4)	0.388	0.7 (0.3-2.0)	0.562
- Downgrading of services and delivery (% yes)	4.8 (0.5-45.9)	0.173	1.0 (0.3-3.2)	0.978	4.9 (0.4-61.9)	0.221

The table only shows variables with p ≤ 0.2. For the within-group analysis, Adjusted Odds Ratio (AdjOR) > 1.0 favours increase from baseline to follow-up. For the between-group analysis, Ratio Odds Ratio (ROR) > 1.0 favours intervention.

^1 ^N is the no of participants who returned completed questionnaires. The N in the four samples varied for each question due to missing answers on the variables (0%-8.2%).

* p-value < 0.1

** p-value < 0.05

# Questions marked ^# ^were added to the Consumer Participation Questionnaire (CPQ) in this study.

^† ^p-value calculated using logistic regression and test of proportions.

### Comparison between the groups

Comparing the changes in the intervention group with the changes in the control group revealed no statistically significant differences at a 0.05 level (table 4 third column). The implementation of the development plan thus had no measurable effect on the professionals at the intervention hospital compared to the control hospitals, neither on knowledge, practice, or attitudes towards user participation.

#### Knowledge about user participation

For knowledge about user participation there was a trend for difference on question no. 11, "Users are invited to participate in professionals' training meetings" which showed an adjusted OR of 4.4 (p = 0.098) in favour of the intervention. Looking for any direction of difference between the groups, the comparisons on knowledge showed a ratio odds ratio (ROR) above 1.0 (favour intervention) on six out of 10 questions (p = 0.098-0.902).

#### Practice of user participation

For practice of user participation the comparisons showed a ROR above 1.0 (favour intervention) on one out of seven questions (p = 0.627).

#### Attitudes towards user participation

For attitudes towards user participation there was a trend for difference on the question no. 6, "Where does the responsibility for deciding the goals of treatment usually lie?" which showed an adjusted OR of 3.3 (p = 0.082) favouring the intervention. Regarding attitudes towards user participation the comparisons show a ROR above 1.0 (favour intervention) on five out of 8 questions (p = 0.082-0.583).

## Discussion

The main result from this study was that implementing a development plan intending to enhance users' participation in a mental health hospital had no significant effect on the professionals' knowledge, practice, or attitudes towards user participation.

### Strengths and limitations

A major limitation in this study is that the samples were not matched, due to maintaining the participants' anonymity. The lack of matching could have provided different samples at the two time periods, and therefore influence the findings considerably. Another limitation is that only professionals were studied. Investigating any changes in the users' experience of user participation would complement the findings. We chose to invite all employees at the hospital to investigate any changes in the organisation as a whole. Including only those working directly with patients might have given different results. Although the study included all three district psychiatric centres in one hospital, providing good representation of professionals in this area, studying professionals in a different area or country could have provided different results. The validation of the questionnaire and the thematic grouping of the questions into knowledge, practice, and attitudes is also a limitation. The thematic grouping was only validated by the researchers based on face validity. This is thus the only validation of whether the questions measure the proposed concepts. There was no further validation to clarify if there are questions that are misplaced or should be omitted in further studies. Furthermore, the sensitivity of the questionnaire to measure changes over time is not known.

A major strength is that the present study is the first non-randomized controlled study investigating the effect on professionals from an extensive and comprehensive development plan intended to enhance users' participation in health care organisation. The overall number of participants was large and the sample was representative for professionals, since the gender distribution was similar to those invited to participate, and the distribution of occupational groups was similar to professionals in mental health hospitals in Central Norway [37]. Although the response rate (55%) was similar to other studies on professionals in mental health services [30,31,38-40], and was similar in the two groups, it is still a possible limitation.

### Challenges in implementing organisational change

Qualitative studies have shown stakeholders' confusion about the purpose of participation, and considerable problems in enhancing participation in health care settings [41,42]. Some state that the general outcome of users participation depends on the way the participation is implemented [43], and that the potential change is influenced by who's initiating the efforts [6]. Although professionals took part in the preparation of the plan in this study, professionals' ownership to the pending change might have been insufficient.

Organisations are often implementing plans intended to make both practical and cultural changes, and most changes are described as failures [44]. Organisations are complex social systems which make cultural change a very difficult task [44,45]. The health care system itself, such as lack of professional power to influence resource priorities and clinical decisions, have also been highlighted as barriers towards user participation [7,46]. Successful changes in organisations have been described as dependent on several steps which have to be undertaken carefully and thoroughly, and where errors in any step might compromise the intended outcome. Successful organisational changes also require a considerable length of time [44], and 16 months might be too short a period of time for any change to manifest, although Storm and colleagues showed impact on some aspects of user involvement only seven months after implementing their intervention program [35]. The other parts of the reorganizational process, i.e. the relocation and merging of several units, might also have overshadowed the focus on enhancing user participation.

### Challenges in evaluating user participation

Some have discussed that undertaking controlled trials on user participation is difficult, since identifying active components and anticipated outcomes is challenging [6]. Some of the initiatives in the development plan were specific, i.e. funding patient education and establishing a patient education centre while others were concerned with more comprehensive cultural and communicational changes in the organisation and among the professionals. Qualitative research has shown that goals on user participation are articulated vaguely and considered implicit by organisations, making it challenging to assess potential outcomes [47].

### Outcome measures for professionals' knowledge, practice and attitudes

The Consumer Participation Questionnaire (CPQ) which was used in this study was developed in New Zealand by Hugh Kent and John Read and has previously been used in cross-sectional studies with some modifications in Wales [31], Canada [38], Austria [40], and Norway [48]. CPQ has been recommended as a mean of measuring the development of user participation in mental health care [30], and to assess professionals' views on user participation in mental health care; both in individual treatment and in planning, delivery and evaluation of mental health services. The CPQ questionnaire has solely been used to measure the existence and amount of user participation in health care organisations, and to compare cross-sectional findings between different settings. It has not previously been used to measure any effects from interventions in controlled studies, and could potentially be unsuitable for such measurements. However; since all professionals in the present study responded to the same questionnaire the comparison between the intervention group and control group will probably not be impacted by these considerations. More testing and validating of this questionnaire is nevertheless needed, e.g due to lack of sensitivity.

## Conclusion

This is the first non-randomized controlled study on the effect of implementing a development plan in mental health hospital to enhance user participation. The plan had no significant effect on professionals' knowledge, practice, or attitudes. This can be due to the quality of the development plan, the implementation process, and/or the suitability of the outcome measures.

## Clinical and research implications

It is not possible to give any advice regarding the implementation of initiatives to enhance user participation based on the results from this study. More controlled studies should be conducted to build further knowledge on the effect on professionals from implementing initiatives to enhance user participation in health care.

## Competing interests

No competing interests have been declared. HG is a member of the board of the hospital trust where the study took place.

## Authors' contributions

MBR designed the study, collected, analyzed and interpreted data, and wrote and completed the manuscript. HG contributed to the interpretation of data, and to the writing and completion of the manuscript. MS designed the study, collected data, and contributed to the writing and completion of the manuscript. AS supervised and designed the study, interpreted data, and contributed to the writing and completion of the manuscript. All authors read and approved the final manuscript.

## Pre-publication history

The pre-publication history for this paper can be accessed here:

http://www.biomedcentral.com/1472-6963/11/296/prepub
